# Comment on Krikorian et al. Early Intervention in Cognitive Aging with Strawberry Supplementation. *Nutrients* 2023, *15*, 4431

**DOI:** 10.3390/nu16060824

**Published:** 2024-03-14

**Authors:** Sang Yeoup Lee

**Affiliations:** 1 Family Medicine Clinic, Biomedical Research Institute, Pusan National University Yangsan Hospital, Yangsan 50612, Republic of Korea; saylee@pnu.edu; Tel.: +82-55-3601442; 2 Integrated Research Institute for Natural Ingredients and Functional Foods, Yangsan 50612, Republic of Korea; 3 Department of Medical Education, Pusan National University School of Medicine, Yangsan 50612, Republic of Korea

I read with interest the paper by Krikorian et al. entitled “Early Intervention in Cognitive Aging with Strawberry Supplementation”, which reports a potential role for strawberry supplementation in reducing dementia risk when started in midlife [1]. The paper addresses the critical issue of dementia—a major public health concern—and emphasizes preventive strategies for cognitive decline beginning in middle age. The unique approach of exploring dietary interventions as an alternative to pharmaceuticals is noteworthy. The strength of this study is further supported by its randomized controlled trial design, which enhances the reliability of the results. Moreover, dietary records and monitoring of flavonoid consumption external to the study solidify the research findings. Nevertheless, it would be beneficial for the authors to provide additional information (outlined below) to facilitate the application of this study in clinical practice.

The study concludes that a 12-week intervention of daily whole-fruit strawberry powder can effectively reduce the risk of dementia in middle-aged individuals with subjective cognitive decline, evidenced by decreased memory interference and depressive symptoms. While there are a few drugs approved to enhance cognition in Alzheimer’s disease (AD), none are approved to protect cognition in patients with mild cognitive impairment [2]. Prior studies have demonstrated cognitive improvements in patients with mild to moderate Alzheimer’s dementia after 12 or 24 weeks [3,4]. The acetylcholine precursor choline alfoscerate showed significant cognitive benefits over placebo after 90 days in patients with mild to moderate AD [3]. Donepezil improved cognitive function from 24 weeks in patients with mild to moderate AD [4].

Interestingly, although the intervention was aimed at patients with subjective cognitive decline rather than diagnosed dementia, cognitive improvement was noted as early as 12 weeks after starting strawberry supplementation. From an evidence-based perspective, a more detailed analysis of the neurocognitive protocol [executive abilities (the Porteus Maze Test, Trail-Making Test, Part B), lexical access (Controlled Oral Word Association Test), learning and long-term memory (California Verbal Learning Test, Second Edition, CVLT; Spatial Paired Associate Learning Test, SPAL), and mood symptoms (Beck Depression Inventory-II)] would be useful for readers. The abstract states consistent improvements in executive abilities among participants treated with strawberries, yet the results section seems to suggest otherwise, creating confusion. To resolve such discrepancies, including comprehensive tables that detail the outcomes of each measure in a clear format is essential.

Firstly, the study does not report statistical values for gender differences in the analysis. The small sample size necessitates the use of Fisher’s exact test to note the difference between groups (*p* = 0.0421). Second, for continuous variables, given the small sample sizes of the groups, it is necessary to validate the normality assumption of the measured variables before proceeding with the statistical analysis. This is particularly relevant for the homeostasis model assessment of insulin resistance (HOMA-IR), which often deviates from a normal distribution, even with larger sample sizes. Third, the study lacks data comparing the differences between the primary outcome variables (total and domain-specific scores) and the secondary outcome variables at the baseline. For example, in Figures 2 and 3, there appears to be a difference in the initial values between the two groups at the start of the study, at week 0. These differences should be adjusted for in the Analysis of Covariance (ANCOVA). The same principle applies to the ANCOVA analysis of the dietary data (see Figure 4) [5]. Fourth, this study omits details on potential side effects or adverse outcomes associated with strawberry supplementation, which are crucial for assessing the intervention’s feasibility and safety. Given that strawberries are the focus of this intervention, a comparison with the effects of other berries high in anthocyanins, such as blueberries, would also be valuable. 

In conclusion, this paper provides new insights into the potential of strawberry supplementation for reducing dementia risk. Nonetheless, a comprehensive presentation of neurocognitive protocol outcomes and more rigorous statistical analysis are required for more convincing results.
